# Epstein–Barr virus and spontaneous lymphoblastoid cell lines: establishment, molecular characteristics, immune modulation, and therapeutic insight

**DOI:** 10.1093/cei/uxaf061

**Published:** 2025-09-15

**Authors:** Adekunle A Adeniran, Gavin Giovannoni, David Baker, Louisa K James

**Affiliations:** Centre for Immunobiology, Blizard Institute, Queen Mary University of London, London, United Kingdom; Centre for Immunobiology, Blizard Institute, Queen Mary University of London, London, United Kingdom; Centre for Immunobiology, Blizard Institute, Queen Mary University of London, London, United Kingdom; Centre for Immunobiology, Blizard Institute, Queen Mary University of London, London, United Kingdom

**Keywords:** spontaneous lymphoblastoid cell line (S-LCL), B-cells, multiple sclerosis (MS), Epstein–Barr virus (EBV), chimeric antigen receptor T-cells (CAR-T)

## Abstract

Epstein–Barr virus (EBV) is a ubiquitous herpesvirus with well-established oncogenic potential, contributing to various malignancies and immune-mediated diseases. Its capacity to infect and immortalize B-cells forms the basis for the generation of lymphoblastoid cell lines (LCLs), which serve as vital models in immunology, virology, and translational research. While conventional LCLs are produced by exogenous EBV infection of peripheral blood mononuclear cells, spontaneous lymphoblastoid cell lines (S-LCLs) can emerge without deliberate viral inoculation, particularly in EBV-seropositive individuals. This review highlights multiple methodologies used to establish S-LCLs, including the use of cyclosporin A, CpG DNA, checkpoint kinase inhibitors, and cytokine modulation, and presents findings from diverse clinical contexts such as autoimmune diseases, post-transplant lymphoproliferative disorders, and cancer. We discuss the biological mechanisms underpinning EBV latency and reactivation, emphasizing the viral transcriptional programmes that drive B-cell transformation and persistence. Additionally, we explore how cytokines, particularly IL-10, support S-LCL survival, and how sodium butyrate and antiviral agents like acyclovir can influence EBV reactivation and replication. The review also considers the diagnostic and therapeutic relevance of LCLs, including their potential as antigen-presenting cells, vaccine platforms, and models for cellular immunotherapies such as CAR T-cells and virus-specific cytotoxic T lymphocytes. By evaluating the generation, molecular features, and immunological significance of S-LCLs, this review underscores their value in modelling EBV-driven disease and advancing novel therapeutic strategies.

## Introduction

The Epstein–Barr virus (EBV), a member of the Herpesviridae family and belonging to the *Lymphocryptovirus* genus, is a lymphotropic virus known for its oncogenic potential. It is highly widespread, with more than 90% of adults worldwide carrying the virus in a latent form [1]. In children, an initial EBV infection typically causes no symptoms or may present as a mild respiratory tract infection [2], but it is known to be the aetiological agent responsible for causing an infection mononucleosis syndrome in the adult population [3]. Beyond acute infection, EBV has been implicated in the development of several malignancies, including Hodgkin’s lymphoma, Burkitt’s lymphoma, nasopharyngeal carcinoma, and post-transplant lymphoproliferative disorders (PTLD), contributing to an estimated 200 000 cancer cases annually [4]. This dual nature of EBV—as both a latent, asymptomatic infection and a driver of oncogenesis—makes it a unique subject of study in immunology, oncology, and virology. In particular, its capacity to infect and transform B-cells provides an invaluable tool for *ex vivo* modelling of immune function and disease. This forms the basis for the generation of LCLs, which have become widely used in research.

Lymphoblastoid cell lines (LCLs) are immortalized B lymphocytes transformed by EBV, characterized by continuous proliferation. These cell lines are generated *in vitro* by infecting human peripheral blood mononuclear cells (PBMCs) with either exogenous or laboratory-adapted strains of EBV. Among these, EBV derived from the B95-8 marmoset cell line is widely used due to its strong capacity for B-cell transformation [5]. The transformation of B-cells by Epstein–Barr virus (EBV) typically occurs within 3–6 weeks following infection. If EBV-infected B lymphocytes do not aggregate into cell clusters by the eighth week, the transformation process is deemed unsuccessful [6]. This failure is primarily attributed to the cytotoxic T-lymphocyte (CTL) response, which targets and eliminates EBV-infected B-cells, reflecting *in vivo* immune surveillance mechanisms.

To overcome this immunological barrier, immunosuppressive agents such as cyclosporin A can be used to inhibit T-cell activity and facilitate successful B-cell immortalization. Alternatively, T-cell mitogens like phytohaemagglutinin activate T-cells, causing them to initially proliferate into blast cells but subsequently undergo apoptosis before maturing into functional cytotoxic T-lymphocytes, thereby temporarily reducing T-cell–mediated cytotoxicity [7]. This interplay between EBV-mediated transformation and host immune responses is critical in determining the efficiency of LCL generation *in vitro* and underscores the complex balance between viral persistence and immune control. Understanding these mechanisms not only informs LCL production protocols but also sheds light on EBV’s pathogenesis and latency control within the host.

EBV is widely employed for B-cell transformation due to its ability to persist in host cells in an episomal form—an extrachromosomal circular DNA—rather than integrating into the germline genome. This episomal persistence results in minimal genetic alterations to the host genome, preserving the integrity of the original genome [8]. Consequently, LCLs serve as a valuable tool for preserving individual-specific cellular characteristics, including their genomic and, to a certain degree, epigenomic profiles. Although some epigenetic markers may be lost during immortalization, many remain intact, making EBV-immortalized cells useful for investigating disease-related epigenomic modifications [9]. The stability and reproducibility of LCLs therefore makes them indispensable tools in genetics, immunology, and biomedical research, providing insights into host-pathogen interactions and the molecular basis of disease.

In addition to the conventional methods of establishing B-cell lymphoblastoid cell lines (B-LCLs) through deliberate EBV infection, S-LCLs have been reported to emerge unexpectedly in cultures derived from myeloid blasts of patients with acute myeloid leukaemia. This phenomenon suggests that S-LCLs can originate *in vitro* from the endogenous B-cell population of EBV-seropositive individuals without the need for exogenous viral inoculation, such as the commonly used B95-8 strain [10]. The spontaneous outgrowth of these cell lines likely reflects *in vivo* viral reactivation or ongoing low-level EBV replication within donor B-cells, which becomes amplified under certain culture conditions. This spontaneous transformation highlights the dynamic nature of EBV latency and reactivation in human hosts and provides a unique window into virus-host interactions that may be especially relevant in disease contexts. For example, our ongoing research explores whether active EBV replication is more pronounced in patients with multiple sclerosis (MS) compared to healthy controls, using S-LCLs as a model system. Investigating these spontaneously derived lines may uncover critical insights into EBV’s role in autoimmune pathogenesis and offer novel opportunities for biomarker discovery and therapeutic targeting.

### Approaches to establishing spontaneous LCLs

#### Production of spontaneous LCLs using cyclosporin A

A study investigating the spontaneous generation of lymphoblastoid cell lines (S-LCLs) explored EBV biology in the context of three distinct groups: individuals with rheumatoid arthritis (RA), patients with infectious mononucleosis (IM), and healthy controls [11]. The primary aim was to assess the capacity of these S-LCLs to express EBV viral antigens and to determine whether antigen expression patterns could reveal strain-specific differences among the groups. By examining these differences, the study sought to shed light on possible associations between EBV strain variability and disease state.

Immunoblot analysis demonstrated that both early and late EBV antigens were detectable in the majority of S-LCLs derived from RA patients and in two of the healthy control lines. Interestingly, while no lytic viral antigen expression was observed in cell lines derived from IM patients, a number of S-LCLs derived from IM patients showed co-expression of distinct antigenic variants, suggesting infection with multiple EBV strains or evidence of intrahost viral diversity. These findings suggest that EBV strain variation may influence the pattern of viral gene expression in spontaneously reactivated infections and could be associated with differing disease outcomes. For instance within the context of disease, there is evidence to show that LMP1 variants from the B95.8 and M81 EBV strains, when expressed in nasopharyngeal epithelial cells induced distinct microRNA expression profiles, demonstrating the role of strain-specific LMP1 sequences in reshaping host cell transcriptomes [12]—potentially leading to different disease outcomes across populations. The EBV sequence variation has been further explored in several recent studies, underscoring both geographic and host-specific diversity. A large-scale analysis combining 217 newly sequenced genomes with over 1300 global datasets found that EBV variation correlates more strongly with geographic origin than with disease type [13]. In Southeast Asia, analysis of EBV genomes from 60 NPC patients of diverse, non-Chinese ancestries revealed ancestry-specific variants, suggesting host–virus co-adaptation and potential ethnic differences in NPC susceptibility [14]. Furthermore, a meta-analysis of 279 NPC cases and 227 controls identified a high-risk EBV lineage marked by a 4-bp EBER2 deletion and a panel of nine SNPs, highlighting its potential utility for NPC risk stratification and screening [15].

The absence of detectable antigen expression in IM-derived lines may reflect strain-specific latency programmes or immune-mediated suppression of viral gene expression. This line of evidence supports the hypothesis that host-pathogen interactions—and potentially EBV strain heterogeneity—play a critical role in modulating EBV activity across different clinical settings.

The S-LCLs were generated by culturing unfractionated mononuclear cells in RPMI-1640 medium supplemented with 0.1 μg/ml of cyclosporin A. Studies have demonstrated that cyclosporin A facilitates the outgrowth of EBV-infected cells by suppressing T-cell-mediated immune responses against the virus [16]. It exerts its immunosuppressive effect by blocking the calcineurin-dependent dephosphorylation of nuclear factor of activated T-cells, preventing its translocation into the nucleus. This inhibition suppresses the transcription of interleukins, particularly IL-2, which is critical for T-lymphocyte activation and differentiation [17, 18].

The cells underwent serial dilution, starting from 2 × 10⁶ down to 1.25 × 10⁵ cells per 0.2 ml well in a microtiter plate. Cyclosporin A was consistently maintained in the culture medium during routine refeeding over an eight-week period, following an already established protocol [19]. Additionally, the culture medium was enriched with 10% foetal calf serum (FCS), and the cells were incubated at 37°C in sealed roller bottles. Although the study did not specify the total number of participants in each group, S-LCLs were successfully generated from seven healthy individuals, seven patients with RA, and seven individuals in the acute phase of IM.

These results demonstrate that sLCL generation is feasible across a range of clinical phenotypes, further supporting the notion that spontaneous B-cell transformation may occur under culture conditions that favour EBV reactivation. This model provides a valuable platform for comparative studies across disease states and could offer insights into differential EBV activity and immune control mechanisms in health versus disease.

#### EBV-transformed LCLs as autologous cancer vaccines

The potential of S-LCLs as alternative antigen-presenting cells (APCs) in cancer vaccine development has been explored, particularly as a means to circumvent major histocompatibility complex (MHC) restrictions [20]. Here, tumour antigen-encoding genes were introduced into S-LCLs, enabling them to present antigens in a broader immunological context. PBMCs were first isolated through Ficoll gradient centrifugation, with 3 × 10⁷ cells plated into 96-well plates at a density of 1 × 10⁶ cells/ml in RPMI 1640 supplemented with 10% FCS. The culture medium was refreshed weekly over a three-week period. Following this, cyclosporin A was introduced at a final concentration of 2 µg/ml. Between 21- and 42-days post-culture initiation, clusters of spontaneously proliferating B-lymphoblastoid cells emerged. These were subcloned into multiple wells, with 20 selected for expansion in 25 cm² cell culture flasks before transitioning to a serum-free medium. The study reported a success rate of approximately 60%, with S-LCLs successfully generated in 9 of 16 healthy donors and 4 of 6 cancer patients.

These findings underscore the promise of S-LCLs not only as models for studying EBV biology but also as functionally relevant APCs for immunotherapeutic applications. Their ease of generation and ability to present antigens without MHC restrictions could represent a cost-effective and scalable alternative to dendritic cells in vaccine development pipelines.

#### Spontaneous LCLs from the B-cells of patients with EBV infection

Studies investigating the molecular features of spontaneously generated S-LCLs have revealed substantial variability in their proliferation rates, particularly in relation to viral microRNA and latent protein expression [3]. The study demonstrated that S-LCLs derived from EBV-infected individuals exhibit divergent growth characteristics, which were closely linked to the expression profiles of EBV-encoded microRNAs and latency-associated proteins. Notably, EBV-infected B-cells from transplant recipients and individuals with IM exhibited highly inconsistent resistance to apoptosis. This suggests that the expansion rate of Type 1 EBV-infected B-cells differs among individuals, ultimately influencing the size of the B-cell reservoir and the likelihood of tumour formation in infected hosts.

To explore these dynamics, the study generated S-LCLs by isolating B-cells from the peripheral blood of three patient groups: immunosuppressed transplant recipients, immunocompetent individuals with IM, and a patient diagnosed with PTLD. A total of 1 × 10⁵ positively selected B-cells were plated onto normal human dermal fibroblast feeder cells in 96-well plates. The cultures were maintained in RPMI-1640 medium supplemented with 10% foetal bovine serum [3]. Overall, fourteen S-LCLs were successfully established from thirteen immunosuppressed transplant recipients, ten S-LCLs were derived from nine immunocompetent patients with IM, and a single S-LCL was obtained from the patient with PTLD.

These results underscore the influence of host immune status and viral gene expression on the transformation outcome. The heterogeneity observed in S-LCL proliferation and apoptosis resistance further suggests that intrinsic differences in host–virus interactions may shape disease susceptibility and progression, particularly in immunocompromised individuals.

#### Contribution of IL-10 in the establishment of S-LCLs

It has been demonstrated that S-LCLs can be successfully established from patients diagnosed with EBV-associated lymphoproliferative disorders without the need for exogenous growth factors or additional viral inoculation. Here, S-LCLs were derived from four such patients, highlighting their capacity to emerge naturally under appropriate culture conditions. These S-LCLs intrinsically secrete a range of cytokines, including IL-6, IL-10, TNF-α, and lymphotoxin-α, reflecting an active autocrine and paracrine signalling environment. Among these cytokines, IL-10 was identified as the key mediator exerting a significant autocrine effect on S-LCL proliferation and long-term survival [21]—supporting the sustained growth of S-LCLs and likely facilitating immune evasion and viral persistence through immunomodulatory mechanisms. This finding underscores the complex interplay between EBV-driven transformation and cytokine regulation, providing insights into how spontaneous EBV-infected cell lines maintain proliferation and survival independent of external stimuli. Understanding these autocrine loops could inform therapeutic strategies aimed at disrupting EBV-associated lymphoproliferative diseases.

Interleukin-10 (IL-10) also acts as a potent B-cell stimulatory factor—other *in vitro* studies have demonstrated its role in promoting B-cell growth, enhancing differentiation, and increasing survival [22].

Given its dual role in immune modulation and B-cell biology, IL-10 may therefore represent a key factor in both the persistence of EBV and the development of associated malignancies. Understanding the mechanistic involvement of IL-10 in S-LCL formation could therefore offer further insights into how the virus exploits host cytokine networks to drive transformation.

In generating S-LCLs, PBMCs were first isolated via Ficoll gradient centrifugation. 1 × 10⁶ PBMCs were cultured in RPMI-1640 medium supplemented with 10% foetal bovine serum, penicillin (50 U/ml), and streptomycin (50 μg/ml), without the addition of exogenous growth factors, mitogens, or immunosuppressive agents. Cultures were maintained in 96-well plates at 37°C in a 5% CO₂ atmosphere. Small proliferative colonies appeared within two weeks and continued to expand over a period of 4–8 weeks.

Using this approach, 17 distinct S-LCLs derived from 4 transplant recipients presenting with clinical and molecular evidence of EBV-associated lymphoproliferative disorders were successfully established. The ability to generate S-LCLs without external viral inoculation or artificial stimulation underscores their potential as a physiologically relevant model for studying EBV-driven lymphoproliferation in immunocompromised hosts. Furthermore, these cell lines also provide a valuable resource for investigating viral latency, host immune responses, and therapeutic interventions in EBV-associated diseases.

#### Establishing S-LCLs using a modified protocol from the Luftig laboratory

In collaboration with Professor Micah Luftig from the Duke University School of Medicine, we utilized a protocol developed in his laboratory for our investigation into EBV and memory B-cells in MS patients to generate S-LCLs [23]. To achieve this, human PBMCs were isolated from whole blood using Ficoll density gradient centrifugation. The isolated cells were then cultured in T25 flasks with fresh RPMI-1640 medium supplemented with 15% FCS, 1× Penicillin-Streptomycin-Glutamax, and 5 μg/ml Cyclosporin A. The addition of Cyclosporin A was necessary to inhibit T-cell activity, as T-cells within the PBMC fraction could interfere with B-cell proliferation. To enhance transformation efficiency, we included 2.5 μg/ml of CpG DNA (5′TCGTCGTTTTGTCGTTTGTCGT 3′) and 5 μM of a checkpoint kinase 2 inhibitor (Chk2i) [23]. Culture medium was partially replaced on day seven and refreshed weekly thereafter. Since growth rates varied between samples, cultures were assessed under a microscope each week to determine optimal maintenance and progression strategies. Once the cells exhibited clumping, indicating successful transformation, they were transferred to RPMI-1640 supplemented with 10% FCS and antibiotics before being cryopreserved. In a study employing this approach. S-LCLs were established from both MS and non-MS participants, although with varying success rates across cohorts.

### EBV latency and the biology/mechanism of LCL formation

The *in vitro* infection of mature B lymphocytes—such as those derived from umbilical cord blood or peripheral blood lymphocytes—by EBV efficiently induces the transformation of these typically resting, naïve B-cells into actively proliferating lymphoblasts. During this transformation, the infected B-cells express a characteristic set of viral latent proteins that are critical for maintaining viral latency and driving cellular proliferation. These include six Epstein–Barr nuclear antigens (EBNA1, EBNA2, EBNA3A, EBNA3B, EBNA3C, and EBNA-LP) as well as three latent membrane proteins (LMP1, LMP2A, and LMP2B). In addition to these proteins, the transformed cells produce two EBV-encoded non-coding RNAs, EBER1, and EBER2, which are highly abundant and contribute to modulating host immune responses and promoting cell survival [24]. The viral proteins act by mimicking and manipulating host signalling pathways, such as those regulating cell cycle progression and apoptosis, enabling EBV to persist within the host while evading immune detection. Understanding the expression pattern and functions of these latent genes is essential not only for deciphering EBV’s oncogenic potential but also for harnessing transformed lymphoblasts as models in immunological and virological research.

The transformation of naïve B-cells into LCLs is primarily driven by the EBV latency III transcription programme, which activates pathways promoting lymphoblast proliferation and survival. EBNA2 and EBNA-LP are understood to be the first EBV genes expressed following EBV’s infection of resting B-cells [25].

Functional studies using recombinant EBV strains with single-gene mutations identified EBNA2 as the only latent gene essential during early B-cell infection. Within the first eight days, EBNA2 is required to activate naïve B-lymphocytes, promote cell growth and division, and prevent apoptosis. While most latent genes show minimal impact during the pre-latent phase, EBNA2 alone drives the reprogramming of infected B-cells into proliferating lymphoblasts, establishing the foundation for latent infection [26]. Acting as the principal transcriptional regulator during EBV latency, EBNA2 initiates the expression of other EBNAs and LMPs. It also activates the proto-oncogene *MYC* by engaging distal enhancer elements located approximately 400–500 kb upstream, thereby promoting the proliferative capacity of infected B-cells [27].

In vivo, EBV infection triggers the expansion and differentiation of naïve B-cells into proliferating blasts through this latency III growth programme. This stage is characterized by the expression of multiple EBNAs, notably EBNA-3A, EBNA-3B, and EBNA-3C, which collectively regulate the infected cell’s proliferative capacity. These viral proteins contribute to the negative autoregulation of cell proliferation, a crucial process that facilitates the migration of EBV-infected B-cells into the germinal centres of lymphoid tissues. Once localized in the germinal centre, infected cells transition from latency III to latency II transcriptional programmes, reflecting a shift towards a more restricted viral gene expression pattern that supports long-term persistence while evading immune detection [28].

EBV latency is broadly classified into four types—Latency 0, I, II, and III—based on the repertoire of latent gene expression [29]. Latency III, typical of EBV-positive diffuse large B-cell lymphomas and PTLD, represents the most transcriptionally active programme promoting robust B-cell activation, proliferation, and survival, with the expressions of the full complement of latent proteins (EBNA1, EBNA2, EBNA3A, 3B, and 3C, EBNA-LP, LMP1, LMP2A and 2B) [29, 30]. As infected B-cells migrate into the germinal centres, EBV transitions to Latency II, as observed in malignancies such as nasopharyngeal carcinoma and classical Hodgkin lymphoma, and marked by the expression of EBNA1, LMP1, LMP2A, and LMP2B [29, 31].

This dynamic regulation of EBV latency programmes highlights the virus’s sophisticated manipulation of host B-cell biology to establish lifelong infection. Understanding these transcriptional shifts is critical not only for elucidating EBV-driven B-cell transformation but also for exploring how S-LCLs may arise *in vivo*, mimicking stages of viral reactivation and latency transitions observed in natural infections.

During the Latency II phase of EBV infection, infected B-cells receive survival signals that allow them to exit the germinal centre and differentiate into memory B-cells. At this stage, the virus adopts a Latency 0 transcriptional programme, characterized by the complete silencing of viral protein expression in resting memory B-cells. This dormancy facilitates long-term viral persistence by minimizing immune detection. EBV maintains its genome in these cells as episomal circular DNA, avoiding integration into the host genome. Occasionally, when memory B-cells divide, EBV transiently switches to a Latency I programme, marked primarily by the expression of EBNA1, a viral protein essential for maintaining the episome during cell division [30]. This tightly regulated transcriptional control allows EBV to persist lifelong within the host while evading immune clearance.

It is also worth noting that although Latency 0 is defined by the complete absence of viral gene expression, some evidence suggests that residual EBNA1 protein may persist in resting memory B-cells due to its exceptional stability. EBNA1 contains a glycine-alanine repeat domain that limits its proteasomal degradation and impairs antigen presentation, contributing to both immune evasion and long protein half-life, estimated to exceed 20 h in B-cell lines [32, 33].

### Switch from latency to lytic

During latency, EBV persists within the nucleus of infected B-cells in the form of a circular episome, rather than integrating into the host genome. This episomal DNA is chromatinized, associating with host histone proteins and adopting a nucleosomal structure similar to that of cellular chromatin [34], which allows the EBV episome to be regulated epigenetically through histone modifications and DNA methylation, similar to host genes. Understanding the chromatin structure of latent EBV genomes has become increasingly important, not only for deciphering the mechanisms of viral persistence but also for identifying therapeutic targets that might disrupt latency in EBV-associated diseases.

The switch from EBV latency to lytic replication is orchestrated by the coordinated activity of immediate-early (IE) genes, with BZLF1 playing a pivotal role. BZLF1 encodes Zta (also known as Zebra, Z, or EB1), a sequence-specific DNA-binding protein that serves as a master regulator of the lytic cycle. Zta directly binds to the viral origin of lytic replication (ori-Lyt), initiating the cascade that leads to viral DNA replication and productive infection. In addition to its role at ori-Lyt, Zta functions as a transcriptional activator, promoting the expression of its own promoter as well as other IE and early viral genes.

Among the downstream targets of Zta is the BRLF1 gene, which encodes Rta (or R), another critical viral transactivator. Rta synergizes with Zta to amplify the expression of a broader set of lytic genes and ensures the full progression of the lytic programme [35]. Together, Zta and Rta form a regulatory hub that determines whether EBV remains latent or enters productive replication.

The expression of IE lytic genes BZLF1 and BRLF1, which initiate the reactivation of latent EBV, can be induced in B-cells by a range of physiological and chemical stimuli. Among these, engagement of the B-cell receptor (BCR) is a well-documented trigger, mimicking antigen recognition and activating intracellular signalling pathways that promote viral reactivation [36]. In the context of S-LCL formation, such reactivation events may provide the proliferative and survival signals necessary to support transformation and long-term outgrowth of B-cells, even in the absence of experimental infection.

While BZLF1 is central to the latent-to-lytic transition and can be activated by multiple biological and chemical factors, its promoter typically displays low basal activity under resting conditions. However, reactivation can be triggered by external stimuli, particularly those that engage the BCR. Crosslinking the BCR with anti-immunoglobulin (anti-Ig) antibodies activates intracellular signalling cascades that ultimately reach the nucleus, promoting BZLF1 expression and lytic cycle entry—a phenomenon that is primarily demonstrated in EBV-positive Burkitt’s lymphoma cell lines such as Akata [37]. In contrast, lymphoblastoid lines generally do not respond to BCR crosslinking with efficient lytic induction and do not show EBV reactivation [38].

Furthermore, various chemical agents—such as 12-O-tetradecanoylphorbol-13-acetate and calcium ionophores—have been shown to mimic aspects of BCR-mediated signalling, leading to partial or full reactivation of latent EBV. These compounds act downstream of the BCR, suggesting that direct receptor engagement is not always necessary for reactivation; instead, stimulation of key nodes in the signalling network is sufficient [39]. This has significant implications for understanding EBV biology in both health and disease—highlighting how cell-intrinsic or external stimuli, including inflammation or co-infections, could modulate EBV latency.

### What cells form LCLs—naïve, class-switched, or affinity-matured B-cells?

It is well established that the B-cells giving rise to LCLs are those directly infected and transformed by EBV. These cells are typically derived from the pool of resting, naïve B-cells present in peripheral blood prior to viral exposure [40]. Upon EBV infection, these naïve B-cells undergo activation, proliferation, and eventual immortalization, leading to the establishment of continuously growing LCLs. EBV establishes infection in the host by inducing polyclonal activation, proliferation, and transformation of B-cells. During the acute phase, the virus promotes broad B-cell expansion, but in the long term, latency is primarily maintained within the memory B-cell compartment—specifically the IgD⁺CD27⁺ non-switched and IgD⁻CD27⁺ switched memory B-cell subsets. Studies have shown that although EBV does not directly induce immunoglobulin class-switch recombination, it can contribute to somatic hypermutation of immunoglobulin genes [41], likely by mimicking or hijacking components of the germinal centre reaction.

All three major B-cell subsets—naïve, non-switched memory, and class-switched memory B-cells—can be transformed into LCLs *in vitro* following EBV infection. However, studies have shown that successful LCL outgrowth is more commonly driven by the transformation of naïve B-cells. Interestingly, these naïve B-cells often exhibit mutated immunoglobulin genes post-transformation, suggesting that EBV may induce somatic hypermutation or promote the selection of cells with such mutations during *in vitro* culture [41].

### Are there molecular (intracellular) or epigenetic signatures that define LCLs?

The growth of LCLs is driven by the Latency III EBV gene expression programme, often referred to as the LCL growth programme. This programme is characterized by the expression of a full complement of latent viral genes, notably the Epstein–Barr nuclear antigens (EBNAs) and LMPs [31].

S-LCLs are known to secrete a range of cytokines, which can serve as functional markers of successful B-cell transformation and immune activation. Notably, the cytokine expression patterns of LCLs can vary significantly—not only between donors but also among cultures derived from the same individual. This variability is thought to result from a combination of host genetic background, environmental influences, and the dynamic nature of EBV latency and reactivation within B-cells. Despite this heterogeneity, several cytokines are commonly secreted by B-cell–derived LCLs. These include pro-inflammatory and regulatory molecules such as CXCL8 (IL-8), IL-10, IL-13, CXCL10 (IP-10), tumour necrosis factor-alpha (TNF-α), and tumour necrosis factor-beta (TNF-β) [42]. Studying these cytokine profiles may offer insights into disease-specific immune signatures, particularly in contexts where S-LCLs are derived from patients with autoimmune conditions, lymphoproliferative disorders, or chronic viral infections.

Interestingly, recent findings by Delecluse *et al*. (2025) provide new insight into how EBV may actively promote central nervous system (CNS) infiltration in MS. Their study demonstrated that EBV-infected B-cells secrete high levels of the chemokine CCL4, which plays a central role in modulating vascular barrier integrity. Specifically, CCL4 signals through its receptor CCR1 on endothelial cells, activating focal adhesion kinase (FAK) signalling pathways. This signalling cascade induces cytoskeletal remodelling and promotes the upregulation of intercellular adhesion molecule-1 (ICAM-1) on the surface of endothelial cells.

Importantly, these changes facilitate transendothelial migration (diapedesis) of both EBV-infected cells and other immune cells across the vascular endothelium. In *in vivo* models used by Delecluse *et al*. [43], this process led to compromised vascular barriers and enhanced leukocyte trafficking into tissues. Although the study was not MS-specific, the implications for the blood–brain barrier (BBB) are highly relevant.

In the context of MS, where immune infiltration into the CNS is a key feature of disease pathology, CCL4-driven endothelial changes could serve as a critical mechanism by which EBV-infected B-cells contribute to disease propagation. The upregulation of ICAM-1 would enhance leukocyte adhesion and extravasation into the CNS, while FAK-dependent endothelial remodelling could increase BBB permeability. This creates a permissive environment for both EBV-infected B-cells and autoreactive T-cells to enter the CNS, sustaining the inflammatory environment characteristic of MS.

### Cellular therapies—chimeric antigen receptor and cytotoxic T-cells

In the treatment of B-cell-related haematological malignancies, chimeric antigen receptor (CAR) T-cell therapy has emerged as a groundbreaking immunotherapeutic approach. This technique involves isolating T-cells from the patient and genetically engineering them to express synthetic CAR molecules on their surface. These receptors are designed to recognize specific surface antigens expressed on malignant B-cells, thereby redirecting T-cells towards tumour-specific cytotoxicity.

Whilst T-cell therapies have emerged as powerful options in oncology and infection, they remain primarily reserved for relapsed and refractory disease, and not yet considered first-line treatments. For example, the ZUMA-7 trial established axicabtagene ciloleucel (axi-cel) as a standard second-line therapy for patients with early relapsed or refractory large B-cell lymphoma [44]. In this Phase 3 study, axi-cel significantly improved event-free survival compared to the previous standard of care—salvage chemoimmunotherapy followed by autologous stem cell transplantation—marking a major shift in the treatment paradigm for aggressive B-cell lymphomas.

CAR-T therapies targeting B-cell antigens such as CD19 and CD20 have shown remarkable clinical efficacy, leading to durable remissions in patients with otherwise refractory or relapsed B-cell malignancies, including acute lymphoblastic leukaemia (ALL), diffuse large B-cell lymphoma, and chronic lymphocytic leukaemia [45, 46].

Reactivation of latent EBV in immunocompromised individuals, who exhibit diminished activity of virus-specific CTLs, is a significant contributor to morbidity and mortality. This underscores the need for both preventive and therapeutic interventions. Research has shown that genetically modifying EBV-transformed B-cell lines with chimeric adenoviral vectors can effectively generate CTLs that target EBV-, adenovirus-, and cytomegalovirus (CMV)-associated antigens in a single culture system. The infusion of these multispecific CTLs into immunocompromised patients has been linked to viral load reduction and alleviation of virus-related symptoms [47].

Since certain malignancies involve EBV, the presence of CTLs plays a critical role in the immune surveillance of EBV infection by recognizing and eliminating cells expressing latent and lytic EBV antigens. This immune control becomes especially important during episodes of viral reactivation, when EBV gene expression increases, and infected cells become more immunogenic. Importantly, EBV-specific CTLs can be isolated from the peripheral blood of seropositive individuals and expanded *ex vivo*. These CTLs have shown clinical utility in adoptive immunotherapy, particularly in treating EBV-positive PTLD—a serious complication that can arise following organ or stem cell transplantation in immunosuppressed patients [48]. Despite concerns regarding graft-versus-host disease following allogeneic bone marrow transplantation (BMT), several studies have demonstrated that donor leukocyte infusions can induce complete pathological and clinical remission in patients with EBV-associated lymphoproliferative disorders. These therapeutic outcomes are primarily attributed to CTLs within the infused leukocytes that are pre-sensitized to EBV antigens [49].

It is also worth noting that the effect of T-cell therapy on PTLD is limited and results are mixed. Adoptive immunotherapy using EBV-specific cytotoxic T-lymphocytes has however gained traction in the management of relapsed or refractory (R/R) EBV-driven PTLD. Tabelecleucel, the first European Medicines Agency approved off-the-shelf T-cell therapy, offers a non-chemotherapy option, showing ∼50% objective response rates, durable clinical benefit, and low-grade toxicities, making it a viable second-line option after failure of rituximab or immunosuppression reduction [50]. However, broader adoption hinges on addressing cost, access, standardized outcomes, and integration with novel therapies like CAR-T in this vulnerable population.

It should be further noted also that there are no randomized studies available for CAR T-cell therapy patients with EBV associated lymphoma. However, a notable case report describes durable remission following CD19 CAR T-cell therapy in a kidney transplant recipient with refractory PTLD, demonstrating both therapeutic feasibility and graft safety [51]. Nevertheless, as an isolated case, this evidence must be interpreted with caution, given the inherent limitations and potential reporting biases of anecdotal data. These findings underscore the urgent need for prospective clinical trials and larger patient cohorts to establish safety and efficacy.

Similar principles governing the mechanism of T-cell therapies apply to other latent herpesvirus infections, such as CMV. In the context of CMV reactivation, particularly in BMT recipients, CD8⁺ CMV-specific CTLs play a critical role in preventing disease manifestations, including CMV pneumonia. Notably, these CTLs are capable of recognizing and lysing CMV-infected cells in an antigen-independent manner, even in the absence of viral gene expression [52], underscoring the importance of memory T-cell surveillance in controlling latent viral infections and highlighting the therapeutic potential of virus-specific CTLs in adoptive immunotherapy strategies.

The success of such approaches illustrates the broader relevance of understanding host–virus interactions and immune control mechanisms, which are central to both disease pathogenesis and treatment. It also reinforces the utility of models such as LCLs for studying CTL responses to latent viruses like EBV and CMV *in vitro*.

### Treatment with acyclovir and penciclovir

Several researchers propose that EBV plays a crucial role in driving MS disease activity [53, 54].

Recent findings by Soldan *et al*. provide compelling evidence that EBV may not act only as an initiator of MS but also contribute actively to disease progression. Using S-LCLs derived *ex vivo* from MS patients, the study revealed that B-cells from individuals with active disease exhibited increased expression of EBV lytic genes and heightened pro-inflammatory transcriptional signatures. Notably, pharmacological inhibition of EBV replication led to a marked reduction in cytokine production and decreased activation of autologous CD4^+^ T-cells [55]. These results support a model in which dysregulated EBV latency, coupled with intermittent lytic reactivation, drives chronic immune activation in MS. Such findings underscore the potential of targeting EBV biology—particularly lytic phase dynamics—as a novel therapeutic avenue in modulating disease activity and progression. Consequently, targeting EBV through immunotherapeutic strategies, such as CTL therapy, therapeutic vaccines, or antiviral agents—may influence disease progression. To explore this possibility, it is essential to understand the mechanisms of action of these antiviral compounds and assess their effects on B-LCLs.

Acyclovir (ACV) is a synthetic purine nucleoside analogue widely recognized for its antiviral activity against several members of the Herpesviridae family, including herpes simplex viruses Type 1 and 2 (HSV-1, HSV-2), varicella-zoster virus, and EBV, with limited efficacy against CMV [56]. This antiviral agent functions by inhibiting viral DNA synthesis. Its mechanism of action relies on conversion to its active form, ACV triphosphate, through phosphorylation. This process is typically initiated by viral thymidine kinase (TK), followed by further phosphorylation by host kinases. Once activated, ACV triphosphate competes with endogenous deoxyguanosine triphosphate for incorporation into viral DNA, resulting in premature chain termination during replication [57]. Unlike lytic-phase herpesviruses such as HSV, EBV lacks a robust expression of virus-specific TK during latency, leading to minimal phosphorylation of ACV in EBV-infected cells. Despite this limitation, ACV retains some efficacy against EBV due to the high sensitivity of the viral DNA polymerase to the drug [58]. This property makes ACV useful in experimental settings for limiting viral replication and reactivation, including in the context of LCL cultures where controlling EBV activity may be desirable.

Penciclovir, an acyclic guanine derivative, shares structural and functional similarities with ACV. It exerts antiviral activity through a comparable mechanism of action and has a similar spectrum of efficacy [59]. Similar to ACV, penciclovir undergoes selective phosphorylation by the viral TK present within infected cells, converting it into penciclovir monophosphate. This intermediate is subsequently phosphorylated by cellular kinases to form penciclovir triphosphate, the pharmacologically active compound [60]. Penciclovir triphosphate acts as a competitive inhibitor of viral DNA polymerase, thereby blocking viral DNA synthesis and limiting viral replication [61]. Several *in vitro* studies have confirmed penciclovir’s efficacy as a selective inhibitor of EBV, demonstrating its ability to reduce the production of infectious virus particles, suppress viral antigen expression, and inhibit viral DNA replication in cultured cells [62]. These findings highlight penciclovir’s potential as a targeted antiviral agent against EBV, with implications for therapeutic strategies aimed at controlling EBV-associated diseases.

### Treatment with sodium butyrate

A critical aspect of studying the role of EBV in MS disease activity involves the establishment of S—LCLs to facilitate the extraction of DNA and RNA for genome and BCR repertoire analysis. To achieve S-LCL outgrowth without introducing an external EBV strain, it is essential to reactivate the latent virus within the cells. This process triggers the EBV lytic cycle, potentially leading to the infection of additional B-cells in culture, ultimately resulting in the transformation of naïve B-cells. Sodium butyrate, when applied at millimolar concentrations, induces the accumulation of acetylated histone proteins in vertebrate cell lines. This effect is attributed to its function as a histone deacetylase (HDAC) inhibitor, which modulates chromatin structure and gene expression both *in vitro* and *in vivo* [63]. The acetylation of lysine residues in the amino-terminal regions of histones neutralizes their positive charges. This reduction in positive charge weakens the electrostatic interactions between histones and the negatively charged phosphate backbone of DNA. As a result, chromatin structure becomes more relaxed, increasing the accessibility of DNA to transcription factors and the transcriptional machinery. This modification thereby facilitates gene expression by promoting transcription and RNA synthesis [64].

Studies have demonstrated that butyrate derivatives can induce the lytic cycle of EBV in EBV-positive B-cells derived from patients [65]. This induction of viral reactivation is largely attributed to butyrate’s effects on histone modifications and chromatin remodelling, which enhance the accessibility of transcription factors to the promoter region of BZLF1, a key viral gene [66]. The resulting BZLF1 protein is a key regulator of the EBV lytic cycle, functioning as a homodimer that binds to both AP1 sites and Zta response elements in the viral genome, thereby triggering the cascade of gene expression necessary for viral DNA replication and production of infectious virions [67, 68]. This ability of butyrate and similar HDAC inhibitors to reactivate latent EBV highlights the intricate epigenetic regulation governing viral latency and reactivation. This has important implications not only for understanding EBV biology but also for clinical contexts where reactivation might contribute to disease progression or therapeutic resistance.

## Conclusion

LCLs have become indispensable tools for advancing our understanding of EBV biology, host immune responses, and the pathogenesis of EBV-associated diseases. By retaining key genomic and epigenomic characteristics of the original B-cells, LCLs serve as robust models for dissecting host–virus interactions, mechanisms of immune evasion, and potential therapeutic targets. Recent improvements in LCL generation—including spontaneous transformation techniques and the strategic use of immunomodulatory agents—have broadened their applicability across both fundamental research and clinical investigations.

Moreover, the expanding role of LCLs as vaccine delivery vectors and platforms for emerging immunotherapies such as CAR T-cells and cytotoxic T-lymphocytes highlights their critical contribution to developing novel treatments for EBV-driven malignancies and autoimmune disorders. As the molecular dynamics governing LCL biology continue to be elucidated, these models hold promise for unveiling new diagnostic markers and therapeutic avenues, particularly for complex diseases like MS and PTLDs. Continued exploration of LCLs will be essential to harness their full potential in translating EBV research into improved clinical outcomes.

## Data Availability

This review includes summarized data from previously published studies, as well as limited data derived from the authors’ own research. All published data are cited in the reference list. The summarized data from the authors’ study are included within the manuscript. No new datasets were generated or analysed beyond those already presented.

## References

[1] Tzellos S, Farrell P. Epstein-Barr virus sequence variation—biology and disease. Pathogens 2012, 1, 156–74. 25436768 10.3390/pathogens1020156 PMC4235690

[2] Dugan JP, Coleman CB, Haverkos B. Opportunities to target the life cycle of Epstein-Barr virus (EBV) in EBV-associated lymphoproliferative disorders. Front Oncol 2019, 9, 127. 30931253 10.3389/fonc.2019.00127 PMC6428703

[3] Delecluse S, Yu J, Bernhardt K, Haar J, Poirey R, Tsai MH, et al Spontaneous lymphoblastoid cell lines from patients with Epstein-Barr virus infection show highly variable proliferation characteristics that correlate with the expression levels of viral microRNAs. PLoS One 2019, 14, e0222847. 31568538 10.1371/journal.pone.0222847 PMC6768455

[4] Guo R, Gewurz BE. Epigenetic control of the Epstein-Barr lifecycle. Curr Opin Virol 2022, 52, 78–88. 34891084 10.1016/j.coviro.2021.11.013 PMC9112224

[5] Yap HY, Siow TS, Chow SK, Teow SY. Epstein-Barr virus- (EBV-) immortalized lymphoblastoid cell lines (LCLs) express high level of CD23 but low CD27 to support their growth. Adv Virol 2019, 2019, 1–9. 10.1155/2019/6464521 PMC6458955 31049064

[6] Jeon JP . Human lymphoblastoid cell lines in pharmacogenomics. In: Handbook of pharmacogenomics and stratified medicine. London: Elsevier, 2014, 89–110.

[7] Amoli M, Carthy D, Platt H, Ollier W. EBV immortalization of human B lymphocytes separated from small volumes of cryo-preserved whole blood. Int J Epidemiol 2008, 37, i41–5. 18381392 10.1093/ije/dym285

[8] Hussain T, Mulherkar R. Lymphoblastoid cell lines: a continuous in vitro source of cells to study carcinogen sensitivity and DNA repair. Int J Mol Cell Med 2012, 1, 75–87. 24551762 PMC3920499

[9] Gurwitz D . Human lymphoblastoid cell lines: a valuable tool for personalized medicine research. Biochem (Lond) 2016, 38, 6–9.

[10] Hayun M, Szwarcwort M, Rosenberg D, Sahar D, Ofran Y. Spontaneous arising of a lymphoblastoid B-cell line harbouring a pre-leukemic DNMT3A mutation in acute myeloid leukaemia cell culture. J Cellular Molecular Medi 2021, 25, 10778–82. 10.1111/jcmm.16992 PMC8581312 34651440

[11] Sculley TB, Moss DJ, Hazelton RA, Pope JH. Detection of Epstein-Barr virus strain variants in lymphoblastoid cell lines “spontaneously” derived from patients with rheumatoid arthritis, infectious mononucleosis and normal controls. J Gen Virol 1987, 68, 2069–78. 3039039 10.1099/0022-1317-68-8-2069

[12] Müller Coan BG, Cesarman E, Acencio ML, Elgui de Oliveira D. Latent membrane protein 1 (LMP1) from Epstein–Barr virus (EBV) strains M81 and B95.8 modulate miRNA expression when expressed in immortalized human nasopharyngeal cells. Genes (Basel) 2022, 13, 353. 35205397 10.3390/genes13020353 PMC8871543

[13] Briercheck EL, Ravishankar S, Ahmed EH, Carías Alvarado CC, Barrios Menéndez JC, Silva O, et al Geographic EBV variants confound disease-specific variant interpretation and predict variable immune therapy responses. Blood Adv 2024, 8, 3731–44. 38815238 10.1182/bloodadvances.2023012461 PMC11296253

[14] Tee HS, Liang J, Aziz NA, Zhou X, Hisham HA, Tan KE, et al Epstein-Barr virus sequence variations among the understudied nasopharyngeal carcinoma patients of diverse ancestries in Southeast Asia. J Med Virol 2025, 97, e70269. 40042148 10.1002/jmv.70269 PMC11881214

[15] Wong KW, Hui KF, Lam KP, Kwong DL, Lung ML, Yang W, et al Meta-analysis of Epstein-Barr virus genomes in Southern Chinese identifies genetic variants and high risk viral lineage associated with nasopharyngeal carcinoma. PLoS Pathog 2024, 20, e1012263. 38805547 10.1371/journal.ppat.1012263 PMC11161099

[16] Palacios R . Cyclosporin A abrogates proliferation of T cells and generation of suppresser and cytotoxic T-cell function induced by Epstein-Barr virus. Immunobiology 1981, 160, 321–9. 6276295 10.1016/s0171-2985(81)80058-7

[17] Wißfeld J, Hering M, ten Bosch N, Cui G. The immunosuppressive drug cyclosporin A has an immunostimulatory function in CD8^+^ T cells. Eur J Immunol 2024, 54, 2350825. 10.1002/eji.202350825 38650034

[18] Tapia C, Nessel TA, Zito PM. Cyclosporine. In: StatPearls. StatPearls Publishing, 2025. Accessed July 4, 2025. http://www.ncbi.nlm.nih.gov/books/NBK482450/29494057

[19] Rickinson AB, Rowe M, Hart IJ, Yao QY, Henderson LE, Rabin H, et al T-cell-mediated regression of “spontaneous” and of Epstein-Barr virus-induced B-cell transformation in vitro: studies with cyclosporin A. Cell Immunol 1984, 87, 646–58. 6088089 10.1016/0008-8749(84)90032-7

[20] Kubuschok B, Cochlovius C, Jung W, Schmits R, Trümper L, Hartmann F, et al Gene-modified spontaneous Epstein-Barr virus-transformed lymphoblastoid cell lines as autologous cancer vaccines: mutated p21 ras oncogene as a model. Cancer Gene Ther 2000, 7, 1231–40. 11023195 10.1038/sj.cgt.7700236

[21] Beatty PR, Krams SM, Martinez OM. Involvement of IL-10 in the autonomous growth of EBV-transformed B cell lines. The Journal of Immunology 1997, 158, 4045–51. 9126962

[22] Go NF, Castle BE, Barrett R, Kastelein R, Dang W, Mosmann TR, et al Interleukin 10, a novel B cell stimulatory factor: unresponsiveness of X chromosome-linked immunodeficiency B cells. J Exp Med 1990, 172, 1625–31. 2124252 10.1084/jem.172.6.1625 PMC2188770

[23] Nikitin PA, Yan CM, Forte E, Bocedi A, Tourigny JP, White RE, et al An ATM/Chk2-mediated DNA damage-responsive signaling pathway suppresses Epstein-Barr virus transformation of primary human B cells. Cell Host Microbe 2010, 8, 510–22. 21147465 10.1016/j.chom.2010.11.004 PMC3049316

[24] Prang NS, Hornef MW, Jäger M, Wagner HJ, Wolf H, Schwarzmann FM. Lytic replication of Epstein-Barr virus in the peripheral blood: analysis of viral gene expression in B lymphocytes during infectious mononucleosis and in the normal carrier state. Blood 1997, 89, 1665–77. 9057649

[25] Alfieri C, Birkenbach M, Kieff E. Early events in Epstein-Barr virus infection of human B lymphocytes. Virology 1991, 181, 595–608. 1849678 10.1016/0042-6822(91)90893-g

[26] Pich D, Mrozek-Gorska P, Bouvet M, Sugimoto A, Akidil E, Grundhoff A, et al First days in the life of naive human B lymphocytes infected with Epstein-Barr virus. mBio 2019, 10, e01723-19. 31530670 10.1128/mBio.01723-19 PMC6751056

[27] Zhao B . Epstein–Barr virus B cell growth transformation: the nuclear events. Viruses 2023, 15, 832. 37112815 10.3390/v15040832 PMC10146190

[28] Price AM, Tourigny JP, Forte E, Salinas RE, Dave SS, Luftig MA. Analysis of Epstein-Barr virus-regulated host gene expression changes through primary B-cell outgrowth reveals delayed kinetics of latent membrane protein 1-mediated NF-κB activation. J Virol 2012, 86, 11096–106. 22855490 10.1128/JVI.01069-12 PMC3457162

[29] Wen KW, Wang L, Menke JR, Damania B. Cancers associated with human gammaherpesviruses. FEBS J 2022, 289, 7631–69. 34536980 10.1111/febs.16206 PMC9019786

[30] Young LS, Rickinson AB. Epstein-Barr virus: 40 years on. Nat Rev Cancer 2004, 4, 757–68. 15510157 10.1038/nrc1452

[31] Price AM, Luftig MA. To be or not IIb: a multi-step process for Epstein-Barr virus latency establishment and consequences for B cell tumorigenesis. PLoS Pathog 2015, 11, e1004656. 25790223 10.1371/journal.ppat.1004656 PMC4366242

[32] Levitskaya J, Sharipo A, Leonchiks A, Ciechanover A, Masucci MG. Inhibition of ubiquitin/proteasome-dependent protein degradation by the Gly-Ala repeat domain of the Epstein-Barr virus nuclear antigen 1. Proc Natl Acad Sci U S A 1997, 94, 12616–21. 9356498 10.1073/pnas.94.23.12616 PMC25057

[33] Apcher S, Daskalogianni C, Manoury B, Fåhraeus R. Epstein Barr virus-encoded EBNA1 interference with MHC class I antigen presentation reveals a close correlation between mRNA translation initiation and antigen presentation. PLoS Pathog 2010, 6, e1001151. 20976201 10.1371/journal.ppat.1001151 PMC2954899

[34] Murata T . Regulation of Epstein–Barr virus reactivation from latency. Microbiol Immunol 2014, 58, 307–17. 24786491 10.1111/1348-0421.12155

[35] Yu X, Wang Z, Mertz JE. ZEB1 regulates the latent-lytic switch in infection by Epstein-Barr virus. PLoS Pathog 2007, 3, e194. 18085824 10.1371/journal.ppat.0030194 PMC2134958

[36] Kenney SC, Mertz JE. Regulation of the latent-lytic switch in Epstein-Barr virus. Semin Cancer Biol 2014, 26, 60–8. 24457012 10.1016/j.semcancer.2014.01.002 PMC4048781

[37] Longnecker R, Miller CL. Regulation of Epstein—Barr virus latency by latent membrane protein 2. Trends Microbiol 1996, 4, 39–42. 10.1016/0966-842x(96)81504-6 8824794

[38] Takada K . Cross-linking of cell surface immunoglobulins induces Epstein-Barr virus in Burkitt lymphoma lines. Intl Journal of Cancer 1984, 33, 27–32. 10.1002/ijc.2910330106 6319296

[39] Murata T, Tsurumi T. Switching of EBV cycles between latent and lytic states. Rev Med Virol 2014, 24, 142–53. 24339346 10.1002/rmv.1780

[40] Mrozek-Gorska P, Buschle A, Pich D, Schwarzmayr T, Fechtner R, Scialdone A, et al Epstein–Barr virus reprograms human B lymphocytes immediately in the prelatent phase of infection. Proc Natl Acad Sci U S A 2019, 116, 16046–55. 31341086 10.1073/pnas.1901314116 PMC6690029

[41] Heath E, Begue-Pastor N, Chaganti S, Croom-Carter D, Shannon-Lowe C, Kube D, et al Epstein-Barr virus infection of Naïve B cells in vitro frequently selects clones with mutated immunoglobulin genotypes: implications for virus biology. PLoS Pathog 2012, 8, e1002697. 22589726 10.1371/journal.ppat.1002697 PMC3349760

[42] Miyauchi K, Urano E, Yoshiyama H, Komano J. Cytokine signatures of transformed B cells with distinct Epstein–Barr virus latencies as a potential diagnostic tool for B cell lymphoma. Cancer Sci 2011, 102, 1236–41. 21392167 10.1111/j.1349-7006.2011.01924.x

[43] Delecluse S, Baccianti F, Zala M, Steffens A, Drenda C, Judt D, et al Epstein–Barr virus induces aberrant B cell migration and diapedesis via FAK-dependent chemotaxis pathways. Nat Commun 2025, 16, 4581. 40389409 10.1038/s41467-025-59813-z PMC12089463

[44] Westin JR, Oluwole OO, Kersten MJ, Miklos DB, Perales MA, Ghobadi A, et al Survival with axicabtagene ciloleucel in large B-cell lymphoma. N Engl J Med 2023, 389, 148–57. 37272527 10.1056/NEJMoa2301665

[45] Grupp SA, Kalos M, Barrett D, Aplenc R, Porter DL, Rheingold SR, et al Chimeric antigen receptor–modified T cells for acute lymphoid leukemia. N Engl J Med 2013, 368, 1509–18. 23527958 10.1056/NEJMoa1215134 PMC4058440

[46] Porter DL, Levine BL, Kalos M, Bagg A, June CH. Chimeric antigen receptor–modified T cells in chronic lymphoid leukemia. N Engl J Med 2011, 365, 725–33. 21830940 10.1056/NEJMoa1103849 PMC3387277

[47] Leen AM, Myers GD, Sili U, Huls MH, Weiss H, Leung KS, et al Monoculture-derived T lymphocytes specific for multiple viruses expand and produce clinically relevant effects in immunocompromised individuals. Nat Med 2006, 12, 1160–6. 16998485 10.1038/nm1475

[48] Haque T, Wilkie GM, Jones MM, Higgins CD, Urquhart G, Wingate P, et al Allogeneic cytotoxic T-cell therapy for EBV-positive posttransplantation lymphoproliferative disease: results of a phase 2 multicenter clinical trial. Blood 2007, 110, 1123–31. 17468341 10.1182/blood-2006-12-063008

[49] Papadopoulos EB, Ladanyi M, Emanuel D, Mackinnon S, Boulad F, Carabasi MH, et al Infusions of donor leukocytes to treat Epstein-Barr virus-associated lymphoproliferative disorders after allogeneic bone marrow transplantation. N Engl J Med 1994, 330, 1185–91. 8093146 10.1056/NEJM199404283301703

[50] Canichella M, de Fabritiis P. Adoptive cell immunotherapy in relapse/refractory Epstein-Barr virus-driven post-transplant lymphoproliferative disorders. Antibodies (Basel) 2025, 14, 47. 40558101 10.3390/antib14020047 PMC12189535

[51] Guy P, Marion O, Oberic L, Darres A, Cointault O, Del Bello A, et al CAR T-cell therapy for refractory posttransplantation lymphoproliferative disorder in a kidney transplant patient. Transplant Direct 2024, 10, e1584. 38414975 10.1097/TXD.0000000000001584 PMC10898664

[52] Riddell SR, Watanabe KS, Goodrich JM, Li CR, Agha ME, Greenberg PD. Restoration of viral immunity in immunodeficient humans by the adoptive transfer of T cell clones. Science 1992, 257, 238–41. 1352912 10.1126/science.1352912

[53] Serafini B, Rosicarelli B, Franciotta D, Magliozzi R, Reynolds R, Cinque P, et al Dysregulated Epstein-Barr virus infection in the multiple sclerosis brain. J Exp Med 2007, 204, 2899–912. 17984305 10.1084/jem.20071030 PMC2118531

[54] Pender MP, Csurhes PA, Smith C, Beagley L, Hooper KD, Raj M, et al Epstein-Barr virus-specific adoptive immunotherapy for progressive multiple sclerosis. Mult Scler 2014, 20, 1541–4. 24493474 10.1177/1352458514521888 PMC4230458

[55] Soldan SS, Su C, Monaco MC, Yoon L, Kannan T, Zankharia U, et al Multiple sclerosis patient-derived spontaneous B cells have distinct EBV and host gene expression profiles in active disease. Nat Microbiol 2024, 9, 1540–54. 38806670 10.1038/s41564-024-01699-6 PMC11900839

[56] O'Brien JJ, Campoli-Richards DM. Acyclovir: an updated review of its antiviral activity, pharmacokinetic properties and therapeutic efficacy. Drugs 1989, 37, 233–309. 2653790 10.2165/00003495-198937030-00002

[57] Shehata H . Drugs and drug therapy. In: Basic science in obstetrics and gynaecology. Edinburgh: Churchill Livingstone, Elsevier, 2010, 259–77.

[58] Pagano JS, Datta AK. Perspectives on interactions of acyclovir with Epstein-Barr and other herpes viruses. Am J Med 1982, 73, 18–26. 6285710 10.1016/0002-9343(82)90057-2

[59] Salvaggio MR, Gnann JW. Drugs for herpesvirus infections. Infect Dis (Auckl) 2017;1:1309–1317.e1.

[60] Aoki FY . Antivirals against herpes viruses. In: Mandell, Douglas, and Bennett’s principles and practice of infectious diseases. Philadelphia, PA: Elsevier Saunders, 2015, 546–562.e7.

[61] Demmler-Harrison GJ . Antiviral agents. In: Feigin and Cherry’s textbook of pediatric infectious diseases. Philadelphia, PA: Elsevier Science (Health Sciences division), 2009, 3245–71.

[62] Bacon TH, Boyd MR. Activity of penciclovir against Epstein-Barr virus. Antimicrob Agents Chemother 1995, 39, 1599–602. 7492112 10.1128/aac.39.7.1599 PMC162789

[63] Candido E . Sodium butyrate inhibits histone deacetylation in cultured cells. Cell 1978, 14, 105–13. 667927 10.1016/0092-8674(78)90305-7

[64] Vidali G, Boffa LC, Bradbury EM, Allfrey VG. Butyrate suppression of histone deacetylation leads to accumulation of multiacetylated forms of histones H3 and H4 and increased DNase I sensitivity of the associated DNA sequences. Proc Natl Acad Sci U S A 1978, 75, 2239–43. 276864 10.1073/pnas.75.5.2239 PMC392527

[65] Ghosh SK, Forman LW, Akinsheye I, Perrine SP, Faller DV. Short, discontinuous exposure to butyrate effectively sensitizes latently EBV-infected lymphoma cells to nucleoside analogue antiviral agents. Blood Cells Mol Dis 2007, 38, 57–65. 17161633 10.1016/j.bcmd.2006.10.00 PMC1829174

[66] Gorres KL, Daigle D, Mohanram S, Miller G. Activation and repression of Epstein-Barr virus and Kaposi’s sarcoma-associated herpesvirus lytic cycles by short- and medium-chain fatty acids. J Virol 2014, 88, 8028–44. 24807711 10.1128/JVI.00722-14 PMC4097796

[67] Chang LK . Activation of the BRLF1 promoter and lytic cycle of Epstein-Barr virus by histone acetylation. Nucleic Acids Res 2000, 28, 3918–25. 11024171 10.1093/nar/28.20.3918 PMC110796

[68] Schepers A, Pich D, Mankertz J, Hammerschmidt W. cis-acting elements in the lytic origin of DNA replication of Epstein-Barr virus. J Virol 1993, 67, 4237–45. 8389925 10.1128/jvi.67.7.4237-4245.1993 PMC237793

